# Assessment of Elbow Joint Incongruity as a Primary Cause of Canine Elbow Dysplasia: Comparative Imaging Analysis Using CT and Radiography in 108 Dogs—A Retrospective Study

**DOI:** 10.3390/life15111673

**Published:** 2025-10-27

**Authors:** Dominika Kubiak-Nowak, Zdzisław Kiełbowicz, Mateusz Hebel, Przemysław Prządka, Agnieszka Antończyk, Marcin Jankowski

**Affiliations:** 1 Department and Clinic of Surgery, Faculty of Veterinary Medicine, Wroclaw University of Environmental and Life Sciences, 50-375 Wroclaw, Poland; zdzislaw.kielbowicz@upwr.edu.pl (Z.K.); mateusz.hebel@upwr.edu.pl (M.H.); przemyslaw.przadka@upwr.edu.pl (P.P.); agnieszka.antonczyk@upwr.edu.pl (A.A.); 2 Department of Internal Medicine with Horse, Dog and Cat Clinic, Faculty of Veterinary Medicine, Wroclaw University of Environmental and Life Sciences, 50-375 Wroclaw, Poland; marcin.jankowski@upwr.edu.pl

**Keywords:** radiology, incongruity, dog, elbow dysplasia

## Abstract

Background: Incongruity is a primary cause of elbow dysplasia. The aims of this study included assessing the prevalence of incongruity, determining reference ranges for joint space widths of the elbow joints in asymptomatic animals, and analyzing the correlations between incongruity and other primary causes of elbow dysplasia. Methods: The study was conducted in 108 dogs of different sexes and aged from 3 to 15 months. The animals were divided into two groups: group I (50 dogs) comprising healthy animals and group II (58 dogs) comprising sick animals. Results: All the dogs in group I were considered healthy. In these dogs, the mean radioulnar “step” height based on the X-ray examination was 0.85 ± 0.33 mm, the mean joint space width of the humeroulnar joint assessed in the computed tomography (CT) examination was 1.34 ± 0.34 mm, and the mean joint space width of the humeroradial joint was 1.43 ± 0.31 mm. In group II, incongruity was detected in 30 and 41 dogs based on the X-ray and CT scan images, respectively. The mean radioulnar “step” height in the dogs with incongruity based on the X-ray examination was 2.05 ± 0.53 mm, while that in the CT examination was 2.33 ± 0.74 mm. The mean joint space width of the humeroulnar joint based on the computed tomography (CT) examination was 3.189 ± 1.03 mm, and the mean joint space width of the humeroradial joint was 2.916 ± 0.702 mm. The most common diagnosis was a combination of incongruity with medial coronoid process disease. Conclusions: Based on the conducted studies, the following reference values were determined: for the height of the radioulnar “step” measured on radiographs, 0.3–1.5 mm; for the height of the radioulnar “step” measured in the CT examination, 0–1.5 mm; for the width of the joint space of the humeroulnar joint measured on MPR images in the sagittal section, 0.8–2.2 mm; and, for the width of the joint space of the humeroradial joint measured on MPR images in the sagittal section, 0.7–2.5 mm. An elbow incongruity was the most frequently diagnosed primary cause of elbow dysplasia, most often occurring in combination with other causes, particularly medial coronoid process disease.

## 1. Introduction

Elbow dysplasia (ED) is a set of changes resulting from one or more primary diseases, including medial coronoid process disease (MCPD) without or with fragmented medial coronoid process (FMCP), ununited anconeal process (UAP), incongruity (IC, INC), and osteochondritis dissecans (OCD). The presence of these conditions leads to the development of irreversible and progressive degenerative joint disease (DJD) [1,2,3,4,5,6]. It is most often diagnosed in young (i.e., aged 4–6 months), rapidly growing dogs of medium, large, and giant breeds. The most predisposed breeds are German Shepherds, Golden Retrievers, Labrador Retrievers, Rottweilers, and Bernese Mountain Dogs [1,7,8,9,10,11,12,13]. In small dog breeds, this disease occurs much less frequently [14]. It has been found that elbow dysplasia occurs twice as often in males than in females, which is likely associated with a rapid growth rate and sex-related factors [1,4,6,15].

Incongruity is considered one of the primary causes of dysplasia, which occurs as a result of uneven bone growth in the elbow joint or abnormal formation of the humeral trochlea, which then takes on a more elliptical shape [5], resulting in widening and misalignment of the joint spaces. Radioulnar (INC R-U), humeroulnar (INC H-U), and humeroradial (INC H-R) incongruity can be found in the elbow joint. In the case of humeroulnar incongruity, the joint space between the trochlear notch of the ulna and the humeral trochlea is widened. Meanwhile, in the case of humeroradial incongruity, the joint space between the radial head and the humeral condyles is widened. It should be noted that humeroradial incongruity often accompanies humeroulnar incongruity. Proximal radioulnar incongruity, in contrast, results from a difference in the level of the articular surface of the radial head in relation to the surface of the lateral coronoid process, leading to the formation of the so-called radioulnar “step” [11,16]. The most common incongruity is humeroulnar incongruity, which is believed to play a role in the pathogenesis of disease of the medial coronoid process due to increased pressure from forces acting on this area during animal movement [13,17,18]. Additionally, incongruity may be associated with the occurrence of a fracture of the medial coronoid process with complete fragmentation, although this is not always observed when diagnosing elbow dysplasia [18].

It should be noted that despite the increasing availability of advanced imaging techniques and numerous studies having been conducted, there are still considerable differences in approaches for determination of the incidence of incongruence as the primary cause of elbow dysplasia, as well as assessments of the elbow joint space width as a diagnostic criterion for this disorder. Therefore, the aims of this study included assessing the frequency of incongruity in dogs of breeds predisposed to elbow dysplasia; determining reference ranges for joint space widths of the humeroulnar, humeroradial, and proximal radioulnar joints in asymptomatic animals; and analyzing the coexistence of incongruity with other primary causes of ED.

## 2. Materials and Methods

### 2.1. Animals

This study was conducted in 108 dogs—including 69 German Shepherds, 23 Labrador Retrievers, and 16 Golden Retrievers—of different sexes (46 males and 62 females) and aged from 3 to 15 months, who were referred to the Imaging Diagnostics Laboratory of the Department and Clinic at the Faculty of Veterinary Medicine, Wroclaw University of Environmental and Life Sciences during the period from November 2017 to December 2019 for elbow imaging procedures.

Exclusion criteria comprised dogs with a history of previous orthopedic surgery; the presence of disorders such as fractures, dislocations, or neoplasia of the elbow joint; dogs aged younger than 3 months or older than 15 months; incomplete medical documentation (missing or incomplete radiographic or CT examinations); and dogs whose owners did not provide consent to participate in the study.

Based on their medical history, clinical examination, and imaging results, the animals were divided into two groups:

Group I: 50 dogs (including 33 German Shepherds, 10 Labrador Retrievers and 7 Golden Retrievers), comprising animals that did not show clinical signs and in which no abnormalities were found on X-ray and computed tomography images.

Group II: 58 dogs (including 36 German Shepherds, 13 Labrador Retrievers and 9 Golden Retrievers), consisting of sick animals (i.e., those showing clinical symptoms and/or abnormalities on X-ray and computed tomography images).

### 2.2. Anesthesia Protocol

Radiographic and computed tomography examinations were performed under sedation consisting of medetomidine (prep. Cepetor, 1 mg/mL, ScanVet Poland (Gniezno, Poland)) at a dose of 10–20 µg/kg bw and butorphanol at a dose of 0.1 mg/kg bw, (prep. Butomidor, 10 mg/mL, ORION PHARMA (Warsaw, Poland)) administered in a single intramuscular injection.

If sedation was insufficient to safely perform the abovementioned examinations, general anesthesia was induced. In addition to premedication, propofol (prep. Propofol-Lipuro, 10 mg/mL, Braun (Melsungen, Germany)) was administered intravenously for induction at an initial dose of 2–4 mg/kg bw, followed by maintenance according to the required effect.

### 2.3. Radiographic Technique

Radiographic examination of the elbow joints in dogs was performed using a Vertix 3D III X-ray (Siemens Healthineers, Erlangen, Germany) machine with a Siemens Polydoros LX 30 lamp (Siemens AG, Erlangen, Germany) and an indirect digital radiography system. The following exposure parameters were applied: lamp voltage from 60 to 70 kV and a product of time and amperage ranging from 6.8 to 7.1 mAs. The setting of these parameters depended on the thickness of the X-rayed tissue. Capturing images did not require the use of an anti-scatter grid.

Radiographs were obtained in 4 projections—(a) mediolateral projection in an extended elbow position, (b) mediolateral projection in flexion, (c) craniocaudal projection, and (d) 15° oblique craniolateral–caudomedial projection—in accordance with the guidelines of the International Elbow Working Group. Each elbow joint was screened separately.

### 2.4. CT Technique

Computed tomography of the elbows was performed using a 16-slice Simens Somatom Emotion CT scanner (Siemens Healthineers, Erlangen, Germany). The examination was performed in dogs in sternal recumbency. To stabilize the animals’ position, a non-shadowing sponge positioner was used. Additionally, the head was tilted to the side and secured with special straps on the CT table. To maximize the exposure of the elbows, the thoracic limbs were pulled out in the cranial direction and placed parallel to each other. Scanning was carried out along the long axis of the elbows, first in the cranial direction and then in the caudal direction. Both elbow joints were scanned simultaneously.

The CT examination of the elbows was performed using the following exposure parameters: 60 mAs and 130 kV; the displacement coefficient was 0.75; the cross-section images were obtained using the bone filter and bone window W 1400, C 300. The image of the elbows was obtained using the Siemens syngoMMWP software, version VE40A. Additionally, software functions were used for multiplanar reconstruction of images in the sagittal, dorsal, and transverse sections and three-dimensional images.

### 2.5. Measurement Techniques

**Figure 1:**
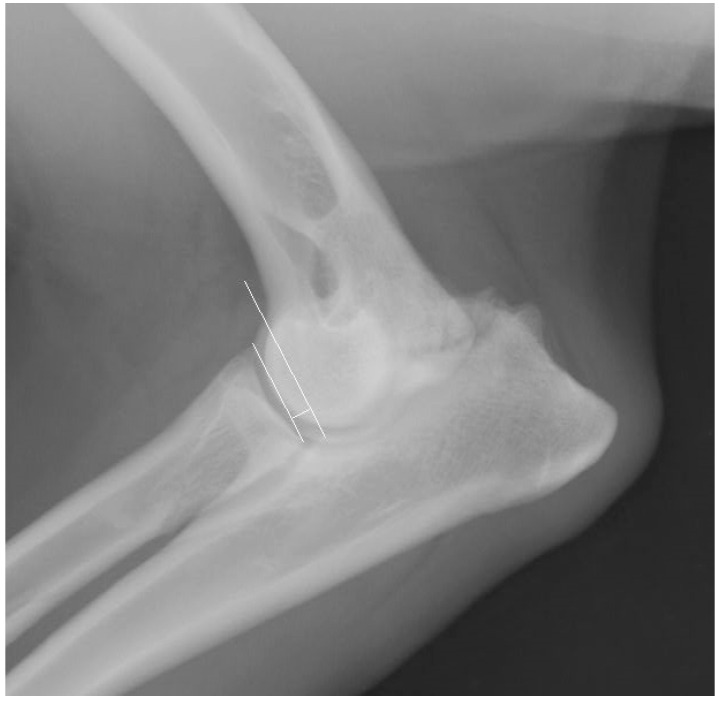
Radiograph obtained in the mediolateral projection in the extended position. Elbow joint dysplasia is visible, along with the measurement of the radioulnar “step” according to the method of Brunnberg et al. (1999) [20].

Based on the obtained radiographs, incongruence was assessed in the mediolateral extension and craniocaudal views according to the criteria proposed by Wind (1986) [19]. The radioulnar “step,” the shape of the trochlear notch of the ulna, the width of the humeroulnar joint space, and the displacement of the humeral condyle were analyzed. The radioulnar “step” was calculated according to the method of Brunnberg et al. [20], which involves marking two lines on the radiograph: the first along the articular surface of the radial head (the so-called radial plateau), and a second parallel line intersecting the tip of the lateral coronoid process (Figure 1). The distance between these lines constituted the radioulnar “step,” which was measured in mm.

**Figure 2:**
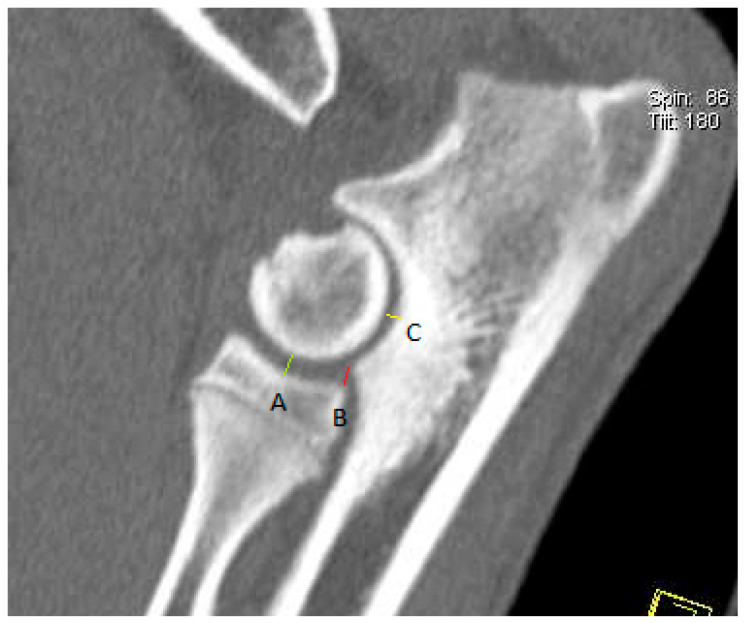
Multiplanar reconstruction computed tomography (CT) image in the sagittal plane. The measurement definitions are shown: width of the humeroradial joint space (A), height of the radioulnar “step” (B), and width of the humeroulnar joint space (C).

**Figure 3:**
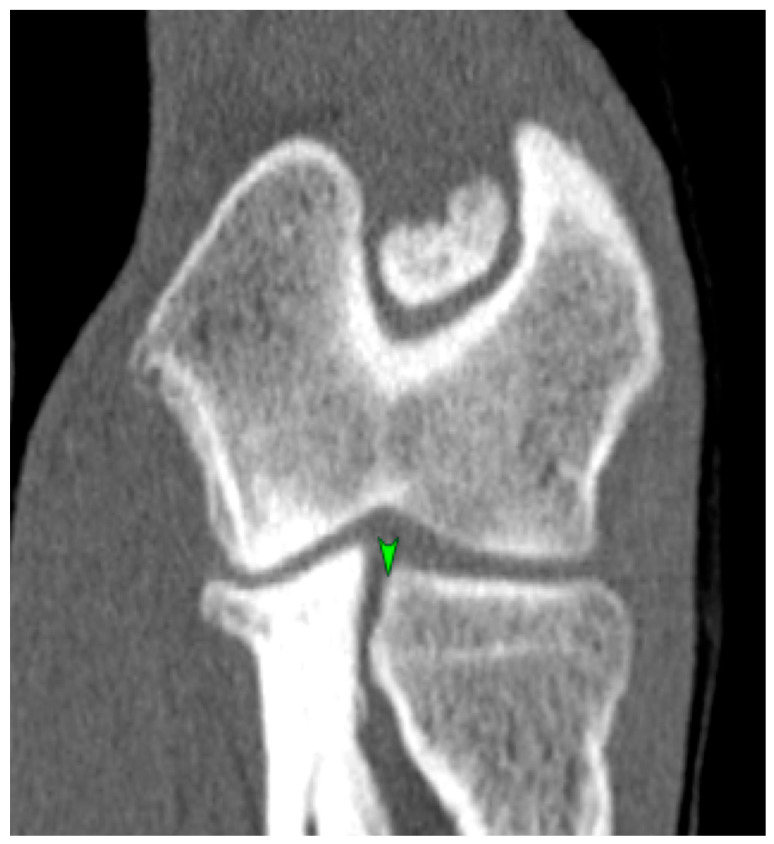
Multiplanar reconstruction computed tomography (CT) image in the dorsal plane showing the measurement site for the height of the radioulnar “step” (green marker).

Each CT image of the elbow joint was analyzed using multiplanar reconstructions in sagittal, dorsal, and transverse sections. The incongruity measurement was performed according to the method proposed by Samoy et al. [18] modified by the authors of this study, which involved measuring the joint space width from the multiplanar reconstruction (MPR) in the sagittal section (Figure 2) and determining the height of the radioulnar “step” in the dorsal section (Figure 3). The joint space width of the humeroulnar joint was measured between the most concave point of the trochlear notch of the ulna and the closest point on the humeral condyle. The joint space width of the humeroradial joint was measured between the most concave point of the radial bone head and the closest point on the humeral condyle. The height of the radioulnar “step” was determined between the most distal point of the trochlear notch of the ulna and the most proximal point of the caudal aspect of the radial head. Additionally, the radioulnar “step” was measured between the axial aspect of the medial coronoid process and the axial aspect of the radial head from the multiplanar view reconstruction in the dorsal section.

In addition to incongruity, the presence of other primary causes of elbow dysplasia was also assessed: medial coronoid process disease, osteochondritis dissecans of the humeral trochlea, and ununited anconeal process. Based on the obtained X-ray and CT images, the frequencies of these conditions and their combinations were determined.

The radiologist who evaluated the radiographs and CT images was blinded to whether each animal belonged to the group of healthy dogs or to the group with elbow dysplasia.

### 2.6. Statistical Analysis

In the statistical analysis of the results obtained, the following tests were employed to assess differences between means:

- Student’s *t*-test for independent samples when comparing two groups;
- One-way analysis of variance (ANOVA) when comparing more than two groups;
- Tukey’s HSD (honestly significant difference) post hoc test for multiple pairwise comparisons, applied following rejection of the hypothesis of equality of all means, to determine between which groups the differences occurred;
- Fisher’s exact test to assess differences in the frequency of occurrence of phenomena between groups (differences between proportions).
- All analyses were conducted at a 5% level of significance using the STATISTICA data analysis software system, version 12 (StatSoft, Inc., Tulsa, OK, USA, 2014), except for Fisher’s exact test, which was performed using the PQStat software, version 1.6.2 (PQStat Software, Poznań, Poland, 2016).

## 3. Results

### 3.1. Test Results of Dogs from Group I

Based on information obtained from the medical history, clinical examination, blood test results, and X-ray and computed tomography of the elbow joints, all the dogs from group I were considered healthy (*n* = 50, 18 males, 32 females). The average age of the animals was 8.23 ± 3.1 months.

#### 3.1.1. X-Ray Results for the Elbow Joints

The mean height of the radioulnar “step”, measured on radiographs in the mediolateral projection in the extended position according to the method of Brunnberg et al. [20], was 0.847 ± 0.33 mm (range: 0.3–1.5 mm). For individual breeds, the values were as follows: in German Shepherds, 0.998 ± 0.25 mm (range: 0.5–1.5 mm); in Labrador Retrievers, 0.595 ± 0.29 mm (range: 0.3–1.4 mm); and in Golden Retrievers, 0.492 ± 0.17 mm (range: 0.3–0.9 mm). Statistically significant differences were found in the mean height of the radioulnar “step” between Labrador Retrievers and German Shepherds (*p* < 0.001) and between Golden Retrievers and German Shepherds (*p* < 0.001). However, no statistically significant difference was found between Labrador Retrievers and Golden Retrievers (*p* = 0.246).

#### 3.1.2. CT Results of the Elbow Joints

**Figure 4:**
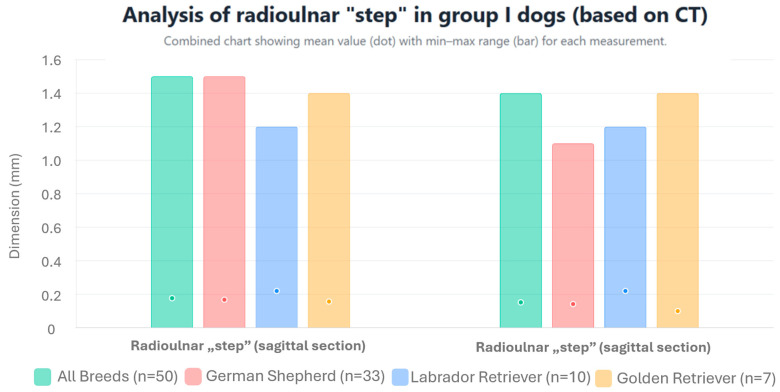
Arithmetic mean, standard deviation, maximum value, and minimum value of the height of the radioulnar “step” in dogs from group I based on CT scans.

In measurements performed on MPR images, in accordance with the method of Samoy et al. [18] modified by the authors of this study, the mean height of the radioulnar “step” was 0.177 ± 0.33 mm (range: 0–1.5 mm) in the sagittal section; meanwhile, in the dorsal section, it was 0.152 ± 0.312 mm (range: 0–1.4 mm). The values of the radioulnar “step” for individual breeds are presented in Figure 4 and Table 1. No statistically significant differences were found in the mean height of the radioulnar “step” in either of the sections (sagittal *p* = 0.810 and dorsal *p* = 0.498) between the studied breeds.

**Figure 5:**
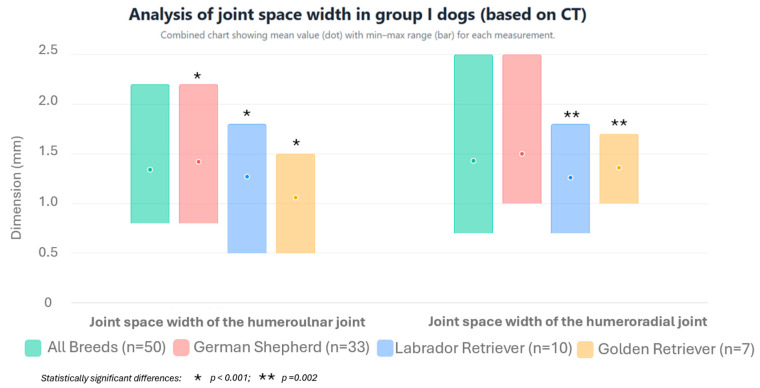
Arithmetic mean, standard deviation, maximum value, and minimum value of the joint space width of the humeroulnar and humeroradial joints in dogs from group I based on CT scans.

The mean joint space width of the humeroulnar joint, measured on MPR images in the sagittal section according to the method of Samoya et al. [18] modified by the authors, was 1.34 ± 0.34 mm (range: 0.8–2.2 mm). For the humeroradial joint, this value was 1.43 ± 0.31 mm (range: 0.7–2.5 mm). The mean joint space width values of the humeroulnar and humeroradial joints for individual breeds are presented in Figure 5 and Table 1. Statistically significant differences were observed in the mean joint space width of the humeroulnar joint between Golden Retrievers and German Shepherds (*p* < 0.001). However, no statistically significant difference was found between Labrador Retrievers and German Shepherds (*p* = 0.070) or Labrador Retrievers and Golden Retrievers (*p* = 0.055). For the humeroradial joint, statistically significant differences were found between Labrador Retrievers and German Shepherds (*p* = 0.002). However, no statistically significant difference was found between Labrador Retrievers and Golden Retrievers (*p* = 0.350) or Golden Retrievers and German Shepherds (*p* = 0.091).

### 3.2. Test Results of Dogs from Group II

#### 3.2.1. X-Ray Results of the Elbow Joints

Based on the elbow radiographs, 38 dogs in group II were diagnosed with elbow dysplasia with a visible primary cause. In 15 dogs from this group, the diagnosis was based on the presence of radiographic signs of secondary degenerative disease without an obvious primary cause. Furthermore, 5 dogs had no radiographic signs of elbow dysplasia.

**Figure 6:**
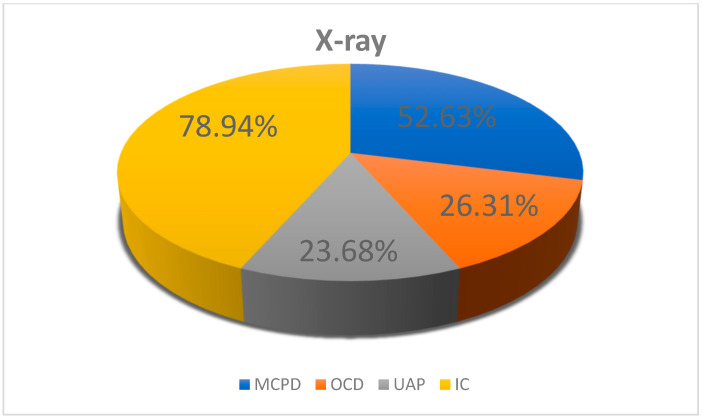
Prevalence of primary causes of elbow dysplasia based on X-ray findings. MCPD, medial coronoid process disease; UAP, ununited anconeal process; OCD, osteochondritis dissecans; IC, incongruity.

Elbow incongruity was present in 30 dogs (including bilateral in 11 cases and unilateral in 19 cases). In 23 dogs, other primary causes of ED were identified (Figure 6). The height of the radioulnar “step,” measured according to the method of Brunnberg et al. [20], ranged from 1.6 to 3.3 mm (mean 2.05 ± 0.53 mm) in the dogs with incongruity; meanwhile, in the dogs from this group without incongruity, this value ranged from 0.2 to 1.5 mm (mean 0.99 ± 0.36 mm). In nine dogs, this measurement was not possible to obtain; furthermore, in one dog, measurement of the height of the radioulnar “step” was not possible and neither a widened humeroulnar joint space, an altered shape of the ulnar trochlear notch, or an altered shape of the humeral trochlea was detected, which was associated with advanced secondary degenerative changes in the elbow joints. Proximal radioulnar incongruity was diagnosed in 28% of cases, an altered shape of the ulnar trochlear notch in 24% of cases, a widened joint space in 19% of cases, and an altered shape of the humeral trochlear notch in only 4% of cases. The frequencies of changes for individual breeds are presented in Table 2.

Statistical analysis revealed significant differences in the mean height of the radioulnar “step” between the dogs from group II with diagnosed incongruity and the dogs from group I (*p* < 0.001), the dogs from group II with diagnosed incongruity and the dogs from group II without incongruity (*p* < 0.001), and the dogs from group I and the dogs from group II without incongruity (*p* = 0.015).

Incongruity was found in 20 German Shepherds, 7 Labrador Retrievers, and 3 Golden Retrievers. The mean height of the radioulnar “step” in German Shepherds was 1.932 ± 0.48 mm, which was statistically significantly lower than that in Labrador Retrievers (2.50 ± 0.51 mm; *p* = 0.028). Statistically significant differences were shown between Labrador Retrievers and German Shepherds (*p* < 0.001) and between Golden Retrievers and German Shepherds (*p* < 0.001); however, no statistically significant differences were found between Labrador Retrievers and Golden Retrievers (*p* = 0.246).

The observed combinations of primary causes of elbow dysplasia are presented in Table 3. Statistically significant differences were observed in the frequencies of primary causes of elbow dysplasia detected individually and in combination (*p* = 0.038).

#### 3.2.2. CT Results for the Elbow Joints

**Figure 7:**
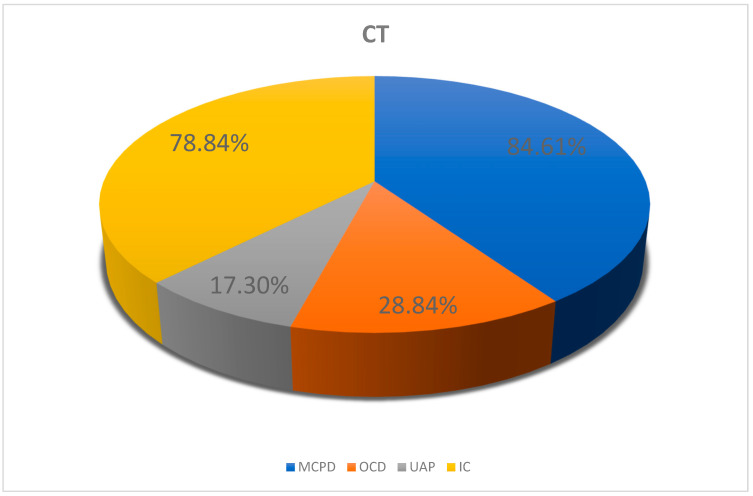
Prevalence of primary causes of elbow dysplasia based on CT findings. MCPD, medial coronoid process disease; UAP, ununited anconeal process; OCD, osteochondritis dissecans; IC, incongruity.

Based on the CT scans of the elbow joints, ED with an apparent primary cause was diagnosed in 52 dogs in group II, while elbow dysplasia without an apparent primary cause was diagnosed in 6 dogs from this group. CT scans showed elbow incongruity in 41 dogs (bilateral in 19 cases, unilateral in 22 cases). However, no incongruity was observed in 17 dogs; in these animals, the cause of elbow joint dysplasia was another primary cause of ED (Figure 7).

The height of the radioulnar “step” in dogs with incongruity, measured from sagittal section MPR images according to the method of Samoy et al. [18] modified by the authors, ranged from 1.6 to 5.2 mm (mean 2.33 ± 0.739 mm). Analogous measurements in the dorsal section ranged from 1.5 to 4.6 mm (mean 2.25 ± 0.758 mm). In the dogs without incongruity on sagittal and dorsal MPR images, these values ranged from 0 to 1.5 mm (mean 0.275 ± 0.504 mm) and 0 to 1.4 mm (mean 0.335 ± 0.550 mm), respectively. Comparing the mean height of the radioulnar “step” measured on sagittal section MPR images, a statistically significant difference was found between the dogs from group II with and without incongruity (*p* < 0.001), as well as between the dogs from group II with diagnosed incongruity and the dogs from group I (*p* < 0.001). Similarly, when comparing the mean height of the radioulnar “step” measured on dorsal section MPR images, a statistically significant difference was found between the dogs from group II with and without incongruity (*p* < 0.001), as well as between the dogs from group II with diagnosed incongruity and the dogs from group I (*p* < 0.001).

The joint space width of the humeroulnar joint in dogs with incongruity, measured on sagittal section MPR images according to the method of Samoy et al. [18] modified by the authors, ranged from 1.0 to 5.8 mm (mean 3.19 ± 1.03 mm). In turn, the joint space width of the humeroradial joint measured on sagittal section MPR images of dogs with incongruity ranged from 1.6 to 4.9 mm (mean 2.92 ± 0.7 mm). In dogs from group II in which no incongruity was detected on sagittal section MPR images, these values ranged from 0.9 to 2.2 mm (mean 1.59 ± 0.41 mm) and 0.7 to 2.5 mm (mean 1.68 ± 0.44 mm), respectively. Comparison of the mean joint space width of the humeroulnar joint measured on sagittal section MPR images revealed a statistically significant difference between the dogs from group II with and without incongruence (*p* < 0.001), as well as between the dogs from group II with diagnosed incongruity and the dogs from group I (*p* < 0.001). Similarly, when comparing the mean width of the joint space of the humeroradial joint measured on sagittal section MPR images, a statistically significant difference was also demonstrated between the dogs from group II with and without incongruity (*p* < 0.001), as well as between the dogs from group II with diagnosed incongruity and the dogs from group I (*p* < 0.001).

**Figure 8:**
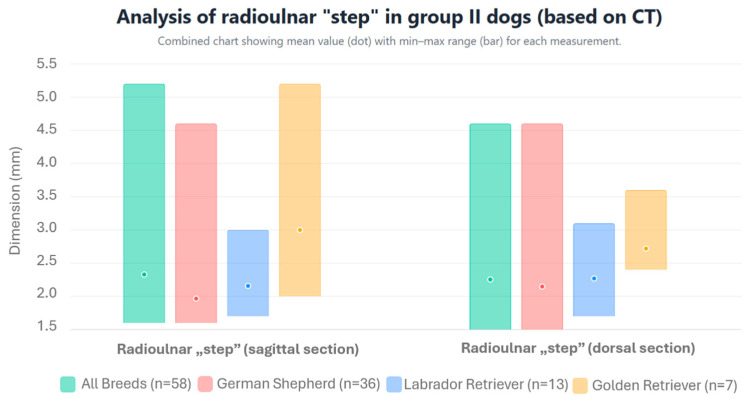
Arithmetic mean, standard deviation, maximum value, and minimum value of the height of the radioulnar “step” in dogs from group II with incongruity based on CT scans.

**Figure 9:**
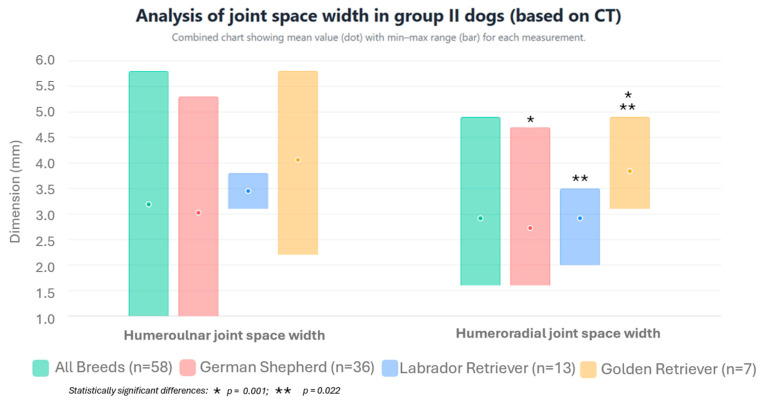
Arithmetic mean, standard deviation, maximum value, and minimum value of the joint space width of the humeroulnar and humeroradial joints in dogs from group II with incongruity based on CT scans.

For individual breeds, incongruity was detected in 29 German Shepherds, which was bilateral in 14 cases and unilateral in 15 cases. In Labrador Retrievers, the disorder was diagnosed in 9 dogs (including 3 bilateral and 6 unilateral cases) and, in Golden Retrievers, incongruity was detected in 3 dogs (including 2 bilateral and 1 unilateral case). The height of the radioulnar “step,” the width of the humeroulnar joint space, and the width of the humeroradial joint space for individual breeds are presented in Figure 8 and Figure 9 and Table 1.

Comparing the mean joint space width of the humeroulnar joint in dogs with diagnosed incongruity, measured on sagittal section MPR images using the method of Samoy et al. [18] modified by the authors, no statistically significant differences were found between the individual breeds (*p* = 0.085). However, when comparing the mean joint space width of the humeroradial joint in dogs with diagnosed incongruity measured on sagittal section MPR images, statistically significant differences were found between Labrador Retrievers and Golden Retrievers (*p* = 0.022) and between Golden Retrievers and German Shepherds (*p* = 0.001), while no statistically significant difference was found between Labrador Retrievers and German Shepherds (*p* = 0.592).

Humeroulnar incongruity was diagnosed in 37% of cases, humeroradial incongruity in 27% of cases, and proximal radioulnar incongruity in 36% of cases. The prevalences of incongruity type by breed are presented in Table 4.

Among the 52 dogs from group II in whom the primary cause of elbow dysplasia could be identified based on computed tomography, incongruity and medial coronoid process disease were the most frequently diagnosed causes. In contrast, osteochondritis dissecans of the medial condyle and ununited anconeal process were significantly less frequently identified. Comparison of the frequencies of primary causes of elbow dysplasia in the dogs from group II diagnosed based on CT revealed statistically significant differences between MCPD and UAP (*p* < 0.001), OCD and IC (*p* < 0.001), UAP and IC (*p* < 0.001), and MCPD and OCD (*p* < 0.001). However, no statistically significant differences were found between MCPD and IC (*p* = 0.675) or OCD and UAP (*p* = 0.252). Among those animals in which the primary cause of ED could be identified based on CT scan, it occurred alone in 12 animals and in combination with other causes in 40 dogs. The combinations of primary causes of ED in the dogs from group II are presented in Table 5.

Comparing the results of the X-ray examination with those of the computed tomography examination in dogs from group II, no statistically significant difference was found regarding the diagnosis of incongruity (*p* = 0.166).

## 4. Discussion

Elbow dysplasia is a disease that often causes reduced motor activity in dogs and is associated with pathological changes in the components of the elbow joint. This results in the following clinical signs: unilateral or bilateral lameness (observed most frequently), elbow pain, swelling, decreased elbow mobility, deformity around the elbow, increased temperature in this area, muscle atrophy, and crepitus observed during flexion and extension of the elbow [1,8,13,21]. In our study, the most common clinical sign observed during the clinical examination in the dogs from group II (dogs exhibiting clinical signs) was lameness, which occurred in all animals, while other clinical signs—such as elbow pain, decreased elbow mobility, deformity around the elbow, and increased temperature in this area—occurred much less frequently. These observations are in line with those reported by other authors [1,13]. It is noteworthy that, in 34 dogs from group II in which the imaging examination diagnosed elbow dysplasia, no clinical signs suggesting this disease were observed. In the authors’ opinion, this was most likely due to the mild stage of the disease and the animals’ adaptation to pain.

In our study, based on changes that are visible on radiographs, incongruity was diagnosed in 78.94% of animals. Proks et al. [22] and Komsta et al. [23] reported slightly lower rates of this primary cause of elbow dysplasia, at 65.18% and 68.91%, respectively. Narojek et al. [14], despite examining 150 dogs, did not detect incongruity in a single case. In the opinion of the authors of this study, determining the height of the radioulnar “step” is very helpful in diagnosing incongruity via X-ray examination. It is difficult to answer the question as to why Narojek et al. [14] did not diagnose this disorder in any case as, in their Materials and Methods, they only provided information on the projections used to perform X-ray examinations of the elbow joints. Notably, they did not describe the method of assessing incongruity, that is, it is not known whether they measured the height of the radioulnar “step,” assessed the width of the joint space of the humeroulnar joint, assessed the shape of the trochlear notch of the ulna, and/or assessed the shape of the trochlea of the humerus [19]. In our own observations, incongruity was most frequently diagnosed in German Shepherds, which accounted for 66.66% of cases. Incongruity was diagnosed significantly less frequently in Labrador Retrievers (23.34% of cases) and Golden Retrievers (10.0% of cases). This disproportion may be due to the larger number of dogs in the German Shepherd group compared with the other two breeds. Remy et al. [24] compared four primary causes of ED in German Shepherds and found incongruity in as many as 90% of cases. In this study, based on the measurement of the radioulnar “step” in animals from group I—in particular, obtained on radiographs in the mediolateral projection in the extended position—reference values for the dogs without incongruity were proposed. The height of the radioulnar “step” in these animals was ≤1.5 mm (mean 0.847 ± 0.33 mm). Comparison of the mean height of the radioulnar “step” in the dogs with incongruity, which was 2.05 ± 0.53 mm, revealed statistically significant differences in relation to the dogs from group I and those from group II which did not show incongruity (mean height of the radioulnar “step” for these dogs: 0.99 ± 0.36 mm). In contrast, Proks et al. [22], based on their observations, concluded that a radioulnar “step” height of 0 to 0.5 mm indicates a normal elbow joint, while a height of this “step” of 0.6 mm and above indicates the presence of incongruity. The differences between our observations and those of Proks et al. [22] may be dependent on the position of the limb for examination of the elbow joint and the angle of the X-ray beam, which has been confirmed in studies performed by other authors [25,26,27,28]. Comparison of the height of the radioulnar “step” in German Shepherds (mean 1.932 ± 0.48 mm) and Labrador Retrievers (mean 2.50 ± 0.51 mm) with diagnosed incongruity showed a statistically significant difference, compared with dogs of these breeds from group I (mean height of the radioulnar “step” for German Shepherds, 0.998 ± 0.25 mm; for Labrador Retrievers, 0.595 ± 0.29 mm).

Our results indicate that CT examination revealed incongruity in 78.84% of the dogs from group II. These observations were in accordance with those of Griffon et al. [29], who diagnosed this disorder in 78.4% of cases. However, Samoy et al. [18] reported a lower incidence of incongruity, finding this disorder in 43.24% of dogs. Among the studied breeds, CT scans revealed incongruity most frequently in German Shepherds. However, the disorder was less common in Labrador Retrievers and Golden Retrievers. Similarly to radiographic examination, the discrepancy in CT findings may have resulted from the unequal representation of dog breeds in the study population, with German Shepherds being the most numerous. Reference values for the parameters considered in this study were determined in the dogs from group I based on measurement of the radioulnar “step” on the multiplanar computed tomography reconstruction images in the sagittal and dorsal sections, as well as measurement of the joint space width of the humeroulnar and humeroradial joints on the sagittal section images. When comparing the height of the radioulnar “step” measured on the multiplanar computed tomography reconstruction images in the sagittal and dorsal sections between the dogs from group I and those with diagnosed incongruity in group II, a statistically significant difference was observed, with the mean values of these heights being 0.117 ± 0.33 mm (sagittal) and 0.152 ± 0.33 mm (dorsal) vs. 2.33 ± 0.739 mm (sagittal) and 2.25 ± 0.758 mm (dorsal). A similar situation was also observed when comparing the joint space width of the humeroulnar and humeroradial joints on multiplanar computed tomography reconstruction images in the sagittal section between the dogs from group I and those diagnosed with incongruity in group II, where the mean values of these parameters were 1.34 ± 0.34 mm (humeroulnar) and 1.43 ± 0.34 mm (humeroradial) vs. 3.19 ± 1.03 mm (humeroulnar) and 2.92 ± 0.7 mm (humeroradial). Comparing the values of the above parameters for German Shepherds, Labrador Retrievers, and Golden Retrievers, statistically significant differences were also revealed between the dogs of these breeds in groups I and II. The above observations are in line with those of Samoy et al. [18], who reported mean heights of the radioulnar “step” in dogs without incongruity measured on multiplanar computed tomography reconstruction images in the sagittal and dorsal sections of 0.21 ± 0.34 mm and 0.31 ± 0.32 mm, respectively, which are very similar to the values obtained in our study. They also demonstrated a statistically significant difference in the height of the radioulnar “step” in affected dogs, where the mean heights on the multiplanar computed tomography reconstruction image in the sagittal and dorsal sections were 3.3 ± 0.93 mm and 3.0 ± 1.0 mm, respectively. Regarding the assessment of the joint space width of the humeroulnar and humeroradial joints measured on the multiplanar computed tomography reconstruction image in the sagittal section, Samoy et al. [18] also found statistically significant differences between healthy dogs and dogs with diagnosed incongruity. The mean values for the joint space width of the humeroulnar and humeroradial joints in healthy dogs were 1.35 ± 0.18 mm and 1.10 ± 0.25 mm, while those for dogs with IC were 3.34 ± 1.15 mm and 3.51 ± 1.05 mm, respectively.

Primary causes of elbow dysplasia may occur alone or in combination [3,5]. In our studies, combinations of primary causes of elbow dysplasia were more common on X-ray and computed tomography (X-ray, 63.16%; CT, 76.93%), compared with primary causes of elbow dysplasia occurring alone (X-ray, 36.84%; CT, 23.07%). In the case of primary causes of elbow dysplasia occurring alone, incongruity was the most common in the X-ray examination (64.3% of cases), while other primary causes of elbow dysplasia were significantly less common (MCPD, 7.11%; UAP, 7.14%; OCD, 21.42% of cases). However, in the CT examination, the most common single primary causes of ED were MCPD (41.66% of cases) and IC (31.35% of cases), while OCD and UAP occurring alone were diagnosed significantly less frequently. Considering the frequency of combinations of primary causes of elbow dysplasia, both the X-ray and CT examinations revealed the combination of MCPD and IC as most frequently diagnosed (X-ray, 45.86%; CT, 55% of cases). The difference in the incidence of combinations of MCPD and IC between X-ray and CT scans—similarly to the frequency of primary causes of ED occurring alone—results from the superior diagnostic utility of CT scans in diagnosing medial coronoid process disease and incongruity. These observations are in line with Remy et al. [30] who reported that, in the case of singular primary causes of ED, MCPD, and IC were the most frequently diagnosed; meanwhile, in the case of combinations of these causes, the combination of MCPD with IC was the most common.

## 5. Conclusions

Based on the conducted research, it was determined that elbow incongruity was the most frequently diagnosed primary cause of elbow dysplasia. Among the studied breeds, this disorder was the most common in German Shepherds; although, it should be noted that this breed was overrepresented in the study population. After analyzing the types of incongruity, it was found that humeroulnar incongruity was the most common. Furthermore, it was observed that incongruity occurred more often in combination with other primary causes of elbow dysplasia than alone, and the most frequent coexisting primary cause of ED was medial coronoid process disease.

Based on the conducted studies, the following reference values were determined: for the height of the radioulnar “step” measured on radiographs, 0.3–1.5 mm; for the height of the radioulnar “step” measured in the CT examination, 0–1.5 mm; for the width of the joint space of the humeroulnar joint measured on MPR images in the sagittal section, 0.8–2.2 mm; and, for the width of the joint space of the humeroradial joint measured on MPR images in the sagittal section, 0.7–2.5 mm. Therefore, in the imaging-based diagnosis of incongruence, both the evaluation of the height of the radioulnar “step” and the width of the joint spaces should be taken into account.

## Figures and Tables

**Table 1:** Arithmetic mean, standard deviation (SD), maximum value, and minimum value of the height of the radioulnar “step” and of the joint space widths of the humeroulnar and humeroradial joints in dogs from groups I and II based on CT scans.

	MPR	Parameter	Unit	Group I				Group II			
				Mean	SD	Maximum	Minimum	Mean	SD	Maximum	Minimum
All breeds	Sagittal section	Joint space of the humeroulnar joint	mm	1.34 *	±0.34	2.2	0.8	3.189 *	±1.03	5.8	1.0
		Joint space of the humeroradial joint	mm	1.43 **	±0.31	2.5	0.7	2.916 **	±0.702	4.9	1.6
		Radioulnar “step”	mm	0.177	±0.333	1.5	0	2.33	±0.739	5.2	1.6
	Dorsal section	Radioulnar “step”	mm	0.152 ***	±0.312	1.4	0	2.255 ***	±0.758	4.6	1.5
German Shepherds	Sagittal section	Joint space of the humeroulnar joint	mm	1.42 ^●^	±0.30	2.2	0.8	3.025	±0.975	5.3	1.0
		Joint space of the humeroradial joint	mm	1.50 ^●●^	±0.30	2.5	1.0	2.725 ^◊^	±0.620	4.7	1.6
		Radioulnar “step”	mm	0.168	±0.321	1.5	0	1.967	±0.673	4.6	1.6
	Dorsal section	Radioulnar “step”	mm	0.142	±0.294	1.1	0	2.148	±0.740	4.6	1.5
Labrador Retrievers	Sagittal section	Joint space of the humeroulnar joint	mm	1.27 ^●^	±0.31	1.8	0.5	3.45	±0.243	3.8	3.1
		Joint space of the humeroradial joint	mm	1.26 ^●●^	±0.31	1.8	0.7	2.917 ^●●●^	±0.611	3.5	2.0
		Radioulnar “step”	mm	0.22	±0.323	1.2	0	2.158	±0.472	3.0	1.7
	Dorsal section	Radioulnar “step”	mm	0.22	±0.322	1.2	0	2.27	±0.472	3.1	1.7
Golden Retrievers	Sagittal section	Joint space of the humeroulnar joint	mm	1.06 ^●^	±0.40	1.5	0.5	4.06	±1.590	5.8	2.2
		Joint space of the humeroradial joint	mm	1.36	±0.25	1.7	1.0	3.84 ^●●●,◊^	±0.713	4.9	3.1
		Radioulnar “step”	mm	0.157	±0.416	1.4	0	3.0	±1.4	5.2	2.0
	Dorsal section	Radioulnar “step”	mm	0.1	±0.374	1.4	0	2.72	±0.68	3.6	2.4

Statistically significant differences: * *p* < 0.001; ** *p* < 0.001; *** *p* < 0.001; ^●^ *p* < 0.001; ^●●^ *p* = 0.002; ^●●●^ *p* = 0.022; ^◊^ *p* = 0.001.

**Table 2:** Prevalence of individual types of incongruity identified via X-Ray.

Breed	Radioulnar “Step”	Joint Space Width of the H–U Joint	Altered Shape of the Trocheal Notch of the Ulnar Bone	Altered Shape of the Humeral Trochlea
German Shepherd Dog	36%	15%	11%	2%
Labrador Retriever	27%	23%	19%	4%
Golden Retriever	0%	27%	27%	11%

**Table 3:** Combinations of primary causes of elbow dysplasia diagnosed based on X-rays.

Combination of Primary Causes of Elbow Dysplasia	Bilateral Elbow Dysplasia		Unilateral Elbow Dysplasia		Number of Cases
	Left Elbow Joint	Right Elbow Joint	Left Elbow Joint	Right Elbow Joint	
**OCD + IC**	-	-	-	OCD + IC	**1** (4.16%)
**OCD + UAP**	OCD + UAP	OCD + UAP	-	-	**2** (8.33%)
**MCPD + UAP**	MCPD + UAP	MCPD + UAP	-	-	**1** (4.16%)
**UAP + IC**	UAP + IC	UAP	-	-	**2** (8.32%)
	-	-	UAP + IC	-	
**MCPD + IC**	MCPD + IC (4)		MCPD + IC (4)	MCPD + IC (3)	**11** (45.89%)
**MCPD + UAP + IC**	MCPD + UAP + IC (1)		MCPD + UAP + IC (1)	MCPD + UAP + IC (1)	**3** (12.5%)
**MCPD + OCD + IC**	MCPD + OCD + IC	MCPD + IC	-	-	**4** (16.64%)
	MCPD + OCD + IC	MCPD + OCD + IC	-	-	
	-	-	MCPD + OCD + IC	-	
	-	-	-	MCPD + OCD + IC	

**Legend:** MCPD—medial coronoid process disease; UAP—ununited anconeal process; OCD—osteochondritis dissecans; IC—incongruity.

**Table 4:** Prevalence of individual types of incongruity identified based on CT.

Breed	INC H-U	INC H-R	INC R-U
German Shepherd	46%	30%	36%
Labrador Retriever	23%	19%	42%
Golden Retriever	22%	27%	27%

**Table 5:** Combinations of primary causes of elbow dysplasia diagnosed based on CT scans.

Combination of Primary Causes of Elbow Dysplasia	Bilateral Elbow Dysplasia		Unilateral Elbow Dysplasia		Number of Cases
	Left Elbow Joint	Right Elbow Joint	Left Elbow Joint	Right Elbow Joint	
**MCPD + IC**	MCPD + IC	IC	-	-	**22** (55%)
	IC	MCPD + IC	-	-	
	MCPD + IC	MCPD + IC	-	-	
	MCPD	MCPD + IC	-	-	
	-	-	-	MCPD + IC	
	-	-	MCPD + IC	-	
**MCPD + OCD + IC**	MCPD + OCD + IC	MCPD + IC	-	-	**9** (22.5%)
	MCPD + IC	MCPD + OCD + IC	-	-	
	MCPD + OCD + IC	MCPD + OCD + IC	-	-	
	MCPD + IC	OCD	-	-	
	-	-	-	MCPD + OCD + IC	
	-	-	MCPD + OCD + IC	-	
**MCPD + OCD**	MCPD + OCD	MCPD	-	-	**1** (2.5%)
**MCPD + UAP + IC**	MCPD + UAP + IC	MCPD	-	-	**4** (10%)
	MCPD + UAP + IC	MCPD + IC	-	-	
	-	-	MCPD + UAP + IC	-	
	-	-	-	MCPD + UAP + IC	
**OCD + UAP**	OCD + UAP	OCD + UAP	-	-	**1** (2.5%)
**MCPD + UAP**	MCPD + UAP	UAP	-	-	**1** (2.5%)
**MCPD + UAP + OCD + IC**	MCPD + UAP + OCD + IC	MCPD + UAP + OCD + IC	-	-	**2** (5%)
	-	-	MCPD + UAP + OCD + IC	-	

**Legend**: MCPD—medial coronoid process disease; UAP—ununited anconeal process; OCD—osteochondritis dissecans; IC—incongruity.

## Data Availability

The original contributions presented in this study are included in the article. Further inquiries can be directed to the corresponding author.

## Abbreviations

The following abbreviations are used in this manuscript:

ED	Elbow dysplasia
DJD	Degenerative joint disease
UAP	Ununited anconeal process
OCD	Osteochondritis dissecans
MCPD	Medial coronoid process disease
FMCP	Fragmented medial coronoid process
INC H-U	Humeroulnar incongruity
INC R-U	Radioulnar incongruity
INC H-R	Humeroradial incongruity
CT	Computed tomography
MPR	Multiplanar reconstruction
SD	Standard deviation
